# Investigating the Neurobiology of Abnormal Social Behaviors

**DOI:** 10.3389/fncir.2021.769314

**Published:** 2021-11-30

**Authors:** S. William Li, Ziv M. Williams, Raymundo Báez-Mendoza

**Affiliations:** ^1^Department of Neurosurgery, Massachusetts General Hospital, Harvard Medical School, Boston, MA, United States; ^2^Department of Anatomy and Neurobiology, Boston University School of Medicine, Boston, MA, United States; ^3^Harvard-MIT Division of Health Sciences and Technology, Boston, MA, United States; ^4^Program in Neuroscience, Harvard Medical School, Boston, MA, United States

**Keywords:** social neuroscience, behavioral neuroscience, animal models, group behavior, abnormal social behavior, psychosocial illness

## Introduction

Social interactions play a crucial role in our daily lives, well-being, and survival. For example, think about the last person you talked to, laughed or shared a meal with, and how it may have affected your mood. Now think about a recent group gathering, event or lab meeting and how it may have affected your actions or even your career. The term “social” is anchored in the processing of information that relates to other individuals and how we interact with them. Humans are social animals, yet many of us suffer from psychiatric illnesses that manifest in symptoms pertaining to social behaviors. These conditions include autism spectrum disorder (ASD), schizophrenia, depression, and social anxiety (Insel and Fernald, 2004; Frith, 2007; Báez-Mendoza et al., 2021b). Despite their prevalence, however, the etiologies behind such deficits are not well-understood and there are few effective treatments for them.

Discovering novel treatments for social deficit disorders requires a fundamental understanding of social behaviors and their neural substrates that are borne not only by observing neurotypical brains but also by describing aberrant behaviors caused by these disorders (Kennedy and Adolphs, 2012). Over the past century, abnormal social behaviors and their neurobiological underpinnings have been studied in humans and animal models, ranging from insects to non-human primates (O'connell and Hofmann, 2012). More complex behaviors have also been reduced to well-defined series of cognitive processes including (1) verbal and non-verbal communication, (2) interpreting others' feelings or intentions, and (3) social interactions. Such divisions have allowed researchers to take advantage of model species that specifically utilize one or more of these behaviors in their natural state, though no animal model can fully describe the complex neurological presentation of social deficit disorders displayed in humans. Nevertheless, many genetic animal models have been created that are well-suited to study certain aspects of these disorders and extrapolate the mechanisms that may underlie such behaviors. Although these models can allow for specific, well-defined phenotypes to be studied in detail, they do not truly capture the complex and multifaceted naturalistic behaviors that define most animal and human behaviors.

## Communicative Behaviors

Successful vocal and non-vocal communication between individuals plays a central role in the social behavior of many animal species (Krause et al., 2009). Communication facilitates the transfer of information between individuals, the identification of individuals or groups, and learning about the animals' environment. In humans, social communication includes verbal and non-verbal components (e.g., social touch, gestures, and facial expressions). Many forms of communicative dysfunction have also been studied across animal species, ranging from erroneous courtship in drosophila (Yost et al., 2020), decreased chemo-signaling in zebrafish and avian species (Caro et al., 2015; Hoffman et al., 2016), atypical ultrasonic vocalizations in rodents (Jamain et al., 2008; Neunuebel et al., 2015; Léna and Mantegazza, 2019), and decreased imitation in the transmission of learned vocalizations in zebra finches (Garcia-Oscos et al., 2021). Additionally, recent development of transgenic non-human primates has culminated in studies finding strikingly similar autism-like verbal communicative dysfunctions in monkey models to human patients (Liu et al., 2016; Zhou et al., 2019). These studies have revealed fundamental neurobiological underpinnings of communicative behaviors and their disorders, including a host of neurocircuit and neurochemical processes involved in a myriad of various behaviors (Chen and Hong, 2018; Tang et al., 2020b).

Although these studies have largely taken advantage of the animals' inherent methods of communicating with one another, reductionist behavioral designs have primarily used either individual or dyadic interactions between animals in confined, artificial environments. While dysfunctional communicative behaviors in patients with social deficit disorders manifest in dyads, they are more prominently displayed in groups of individuals, such as in a classroom or within a sports team (Philip et al., 2012; Lord et al., 2020). Animals show communicative behaviors during group interactions in natural settings. Greeting rituals, where rodents take turns sniffing each other (Wesson, 2013), or where songbirds “tap dance” to each other (Ota et al., 2015), suggest the presence of neuronal representations of conspecifics and the utility of communication. These behaviors shape the animals' future decisions and their social network. Animals also typically forage in groups, utilizing communication to more effectively search for resources (Clark and Mangel, 1986).

Therefore, gaining a full picture of verbal and non-verbal communication must involve behavioral designs that include groups (*n* > 2) of interacting animals. Recent advancements in technology that, for example, can localize and characterize vocalizations in multiple animals (Fonseca et al., 2021) using telemetric technology, automated algorithms, and machine vision, have opened new doors to study communicative behaviors in more naturalistic contexts with groups of animals (Rose et al., 2021). Combining these techniques for automated detection and classification of vocalizations as well as high spatiotemporal resolution marker-less kinematic tracking technologies (Mathis and Mathis, 2020; Topalovic et al., 2020), could broadly expand the types of experiments that mimic social deficit pathology (Banerjee-Basu and Packer, 2010) across avian and mammalian species. While different degrees of complexity evoke distinct social-communicative behaviors and their dysfunction, more naturalistic experimental designs could allow us to gain a better understanding of the interplay between social context (the *where*), agency (the *who*), and the communicative behaviors (the *how*) that underlie social disorders.

## Empathic Behaviors

Empathy refers to the ability of individuals to perceive the internal state of another individual (Smith, 2006) and plays a central role in how we socially interact with others. Individuals with ASD, for example, often struggle relating to the emotions of others, displaying diminished ability to identify the mental states of other individuals or to recognize emotive facial expressions (Baron-Cohen et al., 2000; Lockwood et al., 2016). The past half-century has yielded a golden age of psychosocial experimentations in animals, including non-human primates (Masserman et al., 1964; Bernhardt and Singer, 2012), rats (Church, 1959; Ben-Ami Bartal et al., 2011, 2014), and voles (Burkett et al., 2016), demonstrating the ability of different species to display empathy-like behaviors. These studies have indicated that empathic and prosocial tendencies are conserved across species. Interestingly, these behaviors are augmented when partnered with familiar conspecifics (Silk and House, 2011; Ben-Ami Bartal et al., 2014; Burkett et al., 2016), findings that suggest kin selection as a powerful driver for these phenotypes (Maynard Smith, 1964).

Decreased empathic behaviors have been recently evaluated in animal models using transgenic techniques and neural circuit manipulation. However, little is understood about what specific neural mechanisms related to empathic behavior are disrupted in social behavioral disorders such as ASD. Dysfunction in the medial prefrontal cortex and insula, however, is associated with diminished empathic behaviors in rodents (Rogers-Carter et al., 2018; Lee et al., 2021; Smith et al., 2021), non-human primates (Ballesta and Duhamel, 2015; Gangopadhyay et al., 2021), and humans (Bernhardt and Singer, 2012; Fan et al., 2014). While these studies normally focus on the welfare of all animals involved or animal pairs (Preston and De Waal, 2002; Decety and Svetlova, 2012), it has remained unclear what role specific brain areas or circuits play in ethologically meaningful empathic behaviors or how interindividual differences in personality traits, dominance, or sex affect them. For example, no benefit is associated with helping members of outgroups in some settings, prosocial behaviors may even be maladaptive due to competition for limited resources. Naturally occurring social interactions within groups can also involve empathic behaviors, such as coalition building. Therefore, a better understanding of the neurobiological mechanisms for these behaviors will benefit from longitudinal observations in groups of animals to better capture the group's dynamics (e.g., Rose et al., 2021), and to elucidate the relation between empathy and other social and non-social variables that culminate in strengthening or weakening of group-level behaviors such as social cohesion.

## Interactive Social Behaviors

Social interactions, particularly within groups, play a vital role in the behavior of most animal species and hold broad implications to fields of study in psychology, ecology, evolution, genetics, and neuroscience (Geng and Peterson, 2019; Matthews and Tye, 2019; Mohrle et al., 2020). We recently showed that the prefrontal cortex encodes signals related to specific others' behaviors, a finding only possible when testing the behavior of a group (Báez-Mendoza et al., 2021a). Yet, most of our understanding of social behavior has come from dyadic interactions, which fail to encompass important types of group (or “high-order”) social behavior (Couzin, 2009). While even solitary species display social interactive behaviors such as mating, aggression, and maternal care, species that live in groups display a profoundly more complex social repertoire (Silk and House, 2011). Studies have used standardized assays to quantify sociability and social interactive behavior in animals, such as the three-chamber and the social preference tasks, where interactions are evaluated by pairwise associations (Moy et al., 2004). Dysfunctional social interactions have, therefore, been typically defined by simple metrics such as diminished shoaling in fish (Ogawa et al., 2021), decreased interest in social stimuli in rodents (Lee et al., 2021), and impaired social play in monkeys (Zhou et al., 2019). Gaining a full understanding of social behavior and its underlying neurophysiology, however, requires approaches that access the dynamic interactions among freely behaving individuals and their naturalistic contexts within groups.

The study of naturalistic group behavior has benefited from recent advancements in wireless neuronal recording, inhibition, and stimulation technologies, as well as in computational methodologies that allow tracking the kinematics of multiple animals (Kim et al., 2013; Hultman et al., 2016; Pinti et al., 2018; Anpilov et al., 2020; Berger et al., 2020; Mathis and Mathis, 2020; Topalovic et al., 2020; Marx, 2021). While there is a growing understanding of the behavior of groups (Shemesh et al., 2013; Weissbrod et al., 2013; Harpaz and Schneidman, 2020), there is still little understanding of the neurobiological basis these behaviors (Anpilov et al., 2020; Kim et al., 2020; Tang et al., 2020a). Since animals need to be able to predict the consequences of their behavior on future social interactions in order to make decisions, experimentation of these interactions requires recognition of key social interactions in realistic contexts that captures social and environmental situations that occur in natural habitats (Couzin, 2009). From an evolutionary and ecological perspective, it is essential to relate the cognitive abilities of each species to their social challenges such as their unique environment and group states. Understanding the convergence of these measures upon social decision making, real-time social interactive behaviors, and social affiliation requires an interpretation of higher-level behavioral metrics beyond that of dyadic interactions.

## Discussion

The field of social neuroscience and experimentation in animal models has been extremely fruitful over the past decade, with an increasing emphasis on understanding interactions between pairs of animals under structured task settings. For example, using animal dyads, there has been an expanding understanding of the neural mechanisms and circuits that underlie interactive behaviors such as parenting, social approach, aggression, observational learning, and social bonding. While animal models are well-suited for studying specific, well-defined aspects of social behavior, there is also a need to use naturalistic and ethologically relevant assays that elicit the animals' innate behaviors and environments that organisms rely upon in the wild. More importantly, we need to integrate modeling techniques and experimental paradigms adapted from ecology to better understand the richness and complexity of social behavior and its disruption in psychosocial disease states.

In our search for biomarkers and treatment of social deficit disorders, we need to expand our repertoire of assays and approaches for studying social behavior. Psychosocial disorders often manifest across multiple dimensions including the ability to verbally or non-verbally communicate, interpret the feelings or intentions of others, and effectively interact (Lord and Bishop, 2015). Dysfunctional social behaviors can also be caused by diverse genetic or environmental factors that may differ across individuals and contexts. For example, little attention has been paid to real-world behaviors such as group living or unconstrained naturalistic interactions under which most animal species interact. These limitations, in turn, have made it difficult to interpret and translate data obtained from structured tasks into clinical practice. Therefore, to study and effectively treat these disorders, we need to combine generalizable behavioral measures, naturalistic behavioral paradigms, and telemetric recording techniques. This approach will capture the broader phenomenology of normal and abnormal social behavior. Advancements in social neuroscience will, therefore, likely bring about not only a shift in the way that we quantify social behaviors but also how we observe their neuronal dynamics in both humans and animal models.

## Author Contributions

SWL and RB-M drafted the manuscript. SWL, ZMW, and RB-M revised the manuscript. All authors contributed to the article and approved the submitted version.

## Funding

SWL is supported by the Autism Science Foundation. ZMW is supported by NIH R01HD059852, NIH R01NS091390, and NIH U01NS123130 and RB-M is funded by an MGH-ECOR Fund for Medical Discovery Fellowship and a NARSAD Young Investigator Grant from the Brain and Behavior Research Foundation.

## Conflict of Interest

The authors declare that the research was conducted in the absence of any commercial or financial relationships that could be construed as a potential conflict of interest.

## Publisher's Note

All claims expressed in this article are solely those of the authors and do not necessarily represent those of their affiliated organizations, or those of the publisher, the editors and the reviewers. Any product that may be evaluated in this article, or claim that may be made by its manufacturer, is not guaranteed or endorsed by the publisher.
